# Supplementary material for: Biosynthesis of glucosaminyl phosphatidylglycerol in Pseudomonas aeruginosa
Source: J Bacteriol. 2025 Nov 28;207(12):e00490-24. doi: 10.1128/jb.00490-24 (PMC12713373; doi:10.1128/jb.00490-24)
Supplement: Supplemental figures and table — Figure S1 and S2, and Table S1. [file jb.00490-24-s0001.docx]

**Supplemental Information**

**Figure S1. LC/MS analysis of GlcNAc-PG and GlcN-PG in the PA14_56050 (*gpgF*)** **mutant of *P. aeruginosa***

PA14_56050 (*gpgF*) is bioinformatically predicted to be a lipid flippase. Lipidomic analysis of the PA14 56050::Tn mutant shows that it contains both GlcN-PG and GlcNAc-PG. Compared to WT, the level of GlcN-PG is significantly lower (roughly by several folds), whereas GlcNAc-PG is slightly increased in the PA14 56050::Tn mutant. The presence of GlcN-PG (at a much higher level than GlcNAc-PG) in the *gpgF* mutant suggests that GpgD functions cytosolically, assuming that GpgF is the only flippase for GlcNAc-PG or GlcN-PG.


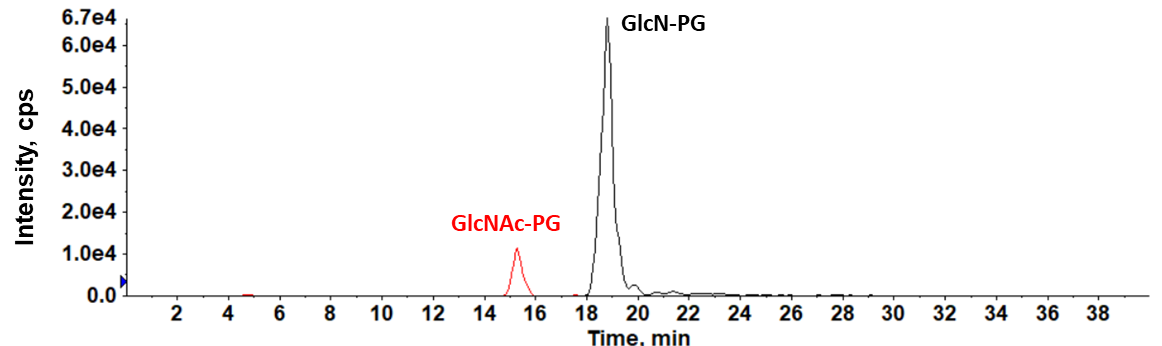


**Figure S2. Heterologous synthesis of GlcNAc-PG in *E. coli* by expressing PA14_56030 (*gpgS*) from *P. aeruginosa***.

A) *E. coli* DH5a lacks both GlcN-PG and GlcNAc-PG.

B) GlcNAc-PG, but not GlcN-PG, is produced by expressing PA14_56050 (GpgS) in *E. coli* DH5a.

C) Negative ion mass spectrum of [M-H]^-^ ions of GlcNAc-PG species produced by expressing PA14_56050 (GpgS) in *E. coli* DH5a.


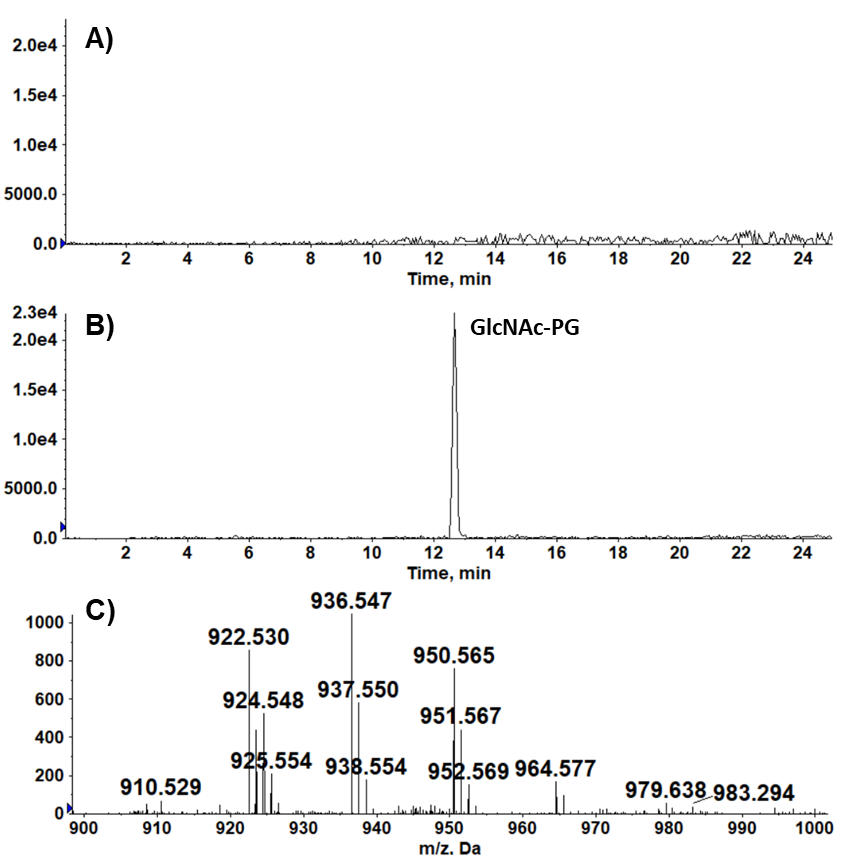


**Table S1. Primers for *gpgD* complementation in PA14 *gpgD*::Tn mutant**

|  | **Primers** | **Sequence (5'-->3') Plasmid-Gene** |
| --- | --- | --- |
| 1 | gpgD_For | CTCATCCGCCAAAACAGCCAATGGCTGAACGCAGCCTGC |
| 2 | gpgD_Rev | CCCGTTTTTTTGGGCTAGCGTCATGCCGCGCGGTCCAG |
| 3 | Screen_pMQ_MCS_For | GCAAATTCTGTTTTATCAGACCGC |
| 4 | pMQ_ScreennRev_1 | GGCGGGTTTCCTCGTCCAGGCCGAGTT |
| 5 | pMQ_ScreenFor_2 | GTCGCCGACGTGCATGTAC |
| 6 | pMQ_ScreenRev_2 | TCAGCCATAGCGCGGGAACC |
| 7 | pMQ_ScreenFor_3 | CACGTCGAGCGCCAGCATGC |
| 8 | pMQ_ScreenRev_3 | CGACGCCAGGCACTGCGC |
| 9 | pMQ_ScreenFor_4 | GAGCGCCGGCACCCGCCAGGC |
| 10 | Screen_pMQ_MCS_Rev | CATAAGATTAGCGGATCCTACCTG |
| 11 | Lin_For_pMQ72 | CGTTGCTGCTCCATAACATCAAAC |
| 12 | Lin_Rev_pMQ72 | TAACAATTCGTTCAAGCCGAGATC |
| 13 | Tetgene_For | GAACGAATTGTTATCAGGTCGAGGTGG |
| 14 | Tetgene_Rev | GGAGCAGCAACGATGAAATCTAACAATG |
| 15 | Tet_scr_pMQ_For | CGCGGCGTTGTGACAATTTAC |
| 16 | Tet_scr_pMQ_Rev | CCAAGCAGCAAGCGCGTTACG |
